# Prokineticin 1 is up‐regulated by insulin in decidualizing human endometrial stromal cells

**DOI:** 10.1111/jcmm.13305

**Published:** 2017-08-07

**Authors:** Dorina Ujvari, Ivika Jakson, Cecilia Oldmark, Sanaz Attarha, Twana Alkasalias, Daniel Salamon, Sebastian Gidlöf, Angelica Lindén Hirschberg

**Affiliations:** ^1^ Department of Women's and Children's Health Karolinska Institutet Stockholm Sweden; ^2^ Department of Obstetrics and Gynecology Karolinska University Hospital Stockholm Sweden; ^3^ Department of Immunology, Genetics and Pathology Uppsala University Uppsala Sweden; ^4^ Department of Microbiology, Tumour, and Cell Biology Karolinska Institutet Stockholm Sweden; ^5^ Department of Biology College of Science Salahaddin University Irbil Kurdistan‐Iraq; ^6^ Department of Clinical Science, Intervention and Technology Karolinska Institutet Stockholm Sweden

**Keywords:** PROK1, EG‐VEGF, insulin, endometrium, decidualization, trophoblast, migration, invasion

## Abstract

Prokineticin 1 (PROK1), a hypoxia‐regulated angiogenic factor, has emerged as a crucial regulator of embryo implantation and placentation. Dysregulation of PROK1 has been linked to recurrent pregnancy loss, pre‐eclampsia, foetal growth restriction and preterm birth. These pregnancy complications are common in women with obesity and polycystic ovary syndrome, i.e. conditions associated with insulin resistance and compensatory hyperinsulinaemia. We investigated the effect of insulin on PROK1 expression during *in vitro* decidualization. Endometrial stromal cells were isolated from six healthy, regularly menstruating women and decidualized *in vitro*. Insulin induced a significant dose‐dependent up‐regulation of PROK1 on both mRNA and protein level in decidualizing endometrial stromal cells. This up‐regulation was mediated by hypoxia‐inducible factor 1‐alpha (HIF1α) *via* the phosphatidylinositol 3‐kinase (PI3K) pathway. Furthermore, we demonstrated that PROK1 did not affect the viability, but significantly inhibited the migration of endometrial stromal cells and the migratory and invasive capacity of trophoblast cell lines. This *in vitro* study provides new insights into the regulation of PROK1 by insulin in human decidualizing endometrial stromal cells, the action of PROK1 on migration of endometrial stromal cells, as well as migration and invasion of trophoblasts. We speculate that hyperinsulinaemia may be involved in the mechanisms by which PROK1 is linked to placenta‐related pregnancy complications.

## Introduction

The tightly coordinated decidual transformation is indispensable for the formation of a functional feto‐maternal interface in order to successfully establish and maintain pregnancy. The central roles of decidualization are the protection of feto‐maternal interface against oxidative stress, local suppression of maternal immune response and control of extravillous trophoblast migration and invasion 1. The latter is mediated, at least in part, by a recently discovered hypoxia‐regulated angiogenic factor PROK1, or alternatively named endocrine gland‐derived vascular endothelial growth factor (EG‐VEGF).

PROK1 has been described in the human endometrium throughout the menstrual cycle in the stroma, glandular epithelium and endothelial cells with increasing levels in the secretory phase 2. Furthermore, PROK1 expression is significantly increased in first trimester decidua, compared with non‐pregnant endometrium 3. PROK1 is expressed in the placenta and is predominantly regulated by oxygen tension. In the hypoxic period of placental development in the first trimester, increased level of placental PROK1 promotes proliferation of anchoring cytotrophoblasts and formation of trophoblastic plugs in the maternal spiral arteries 4, 5. These trophoblastic plugs protect the developing placenta and embryo from highly oxygenated maternal blood and early oxidative damage. Placental PROK1 level declines after week 10–12 of pregnancy as the oxygen tension increases, facilitating deeper invasion of trophoblasts and remodelling of the maternal spiral arteries in order to supply the developing foetus 5, 6. Impaired invasion and thin or fragmented trophoblastic plugs have been associated with early pregnancy loss, while poor invasion of maternal vessels has been correlated to both pre‐eclampsia and foetal growth restriction 4, 6, 7, 8, 9.

PROK1 was shown to be up‐regulated by the hypoxia‐inducible factor 1 (HIF1) in human placenta and human adrenal carcinoma cell lines and by human chorionic gonadotrophin in human placenta 5, 8, 10. HIF1 is a basic helix‐loop‐helix transcription factor composed of two subunits, HIF1α and HIF1β. The latter is constitutively expressed, whereas HIF1α expression is up‐regulated predominantly by hypoxia. Under normoxia, HIF1α is rapidly ubiquitinated by the von Hippel–Lindau tumour suppressor E3 ligase complex and subjected to proteasomal degradation 11. HIF1α was shown to be up‐regulated in luteal phase endometrium in both the stromal and the epithelial cells compared to the proliferative phase of the menstrual cycle 12. It was demonstrated previously in a variety of cell types, but not in endometrial stromal cells, that HIF1α can be regulated not only by hypoxia but also by insulin and other growth factors under normoxic conditions, mainly by up‐regulating HIF1α protein expression or preventing it from proteasomal degradation 13, 14, 15, 16. It was also shown in arising retinal pigment epithelia cell line 19 and primary rat hepatocytes, that HIF1α could be induced by insulin *via* the PI3K pathway 13, 17.

Insulin resistance leading to secondary hyperinsulinaemia has been suggested to be of importance for pregnancy complications including miscarriage, recurrent pregnancy loss and pre‐eclampsia in metabolic disorders such as polycystic ovary syndrome (PCOS) and obesity 18, 19, 20, 21, 22, 23, 24, 25, 26, 27, 28, 29, 30, 31. However, the underlying mechanisms are poorly understood.

We have recently reported data suggesting adverse effect of insulin on endometrial function and *in vitro* decidualization 32, 33. In this *in vitro* study, we aimed to investigate the effect of insulin on the regulation of PROK1 in primary decidualizing human endometrial stromal cells, as well as the effect of PROK1 on migration of human endometrial stromal cells and migration and invasion of trophoblast cells.

## Materials and methods

### Subjects

Endometrial biopsies were collected under local anaesthesia with an endometrial suction curette (Pipet Curet; CooperSurgical, Trumbull, Connecticut, USA) from six regularly cycling, non‐smoking healthy volunteers at cycle day 5–9. All participants were between 18 and 35 years with a body mass index ranging 19–28. Exclusion criteria were hormonal medication within 3 months prior to examination, current chronic disease, endocrine disorder or continuous medication. All women gave their written informed consent, and the Regional Ethical Committee in Stockholm approved the study (Dnr 2008/865‐32).

### Isolation of human endometrial stromal cells

Isolation of endometrial stromal cells was carried out as previously described 33. Purity of stromal cells was ensured by sequential culturing and assessed by cytokeratin and CD10 staining for epithelial and stromal cells, respectively.

### Culture conditions

Endometrial stromal cells were seeded in six‐well Costar plates (Sigma‐Aldrich, St. Louis, Missouri, USA) and cultured in DMEM/F12‐Glutamax (Thermo Fischer Scientific, Waltham, Massachusetts, USA) supplemented with 10% heat‐inactivated foetal bovine serum (HI‐FBS) (Sigma‐Aldrich) and 0.2% penicillin–streptomycin (Sigma‐Aldrich) until ~80% confluency. *In vitro* decidualization was performed with a well‐established procedure, as described previously 33, 34. Briefly, media were changed to phenol red‐free DMEM/F12 (Thermo Fischer Scientific), supplemented with 2% charcoal‐stripped foetal bovine serum (Sigma‐Aldrich) and 0.2% penicillin–streptomycin. To investigate the kinetics of decidualization, we treated the cells with 1 μM medroxyprogesterone‐17‐acetate (MPA) (Sigma‐Aldrich) and 0.5 mM N^6^, 2`‐O‐dibutyryladenosine cAMP (db‐cAMP) (Sigma‐Aldrich) for 3 and 6 days. In order to clarify the contribution of each of MPA and db‐cAMP to induce decidualization, endometrial stromal cells were treated with 1 μM MPA, 0.5 mM db‐cAMP or combined treatment of MPA and db‐cAMP for 6 days. To investigate the effect of insulin on PROK1 during decidualization, cells were treated with 1 μM MPA and 0.5 mM db‐cAMP to induce decidualization for 6 days in the presence or absence of 5, 50 or 500 nM insulin (Sigma‐Aldrich).

To test whether wortmannin, a PI3K inhibitor, could block the effect of insulin, stromal cells were pre‐decidualized for 3 days and then pre‐treated according to previous publications 35, 36 with 500 nM wortmannin (Sigma‐Aldrich) for 1 hr prior to treatment with 100 nM insulin for 2 days in the presence of decidualization agents db‐cAMP and MPA. To evaluate the involvement of HIF1α in the regulation of PROK1 by insulin, stromal cells were pre‐decidualized for 3 days and then pre‐treated with 5 nM of echinomycin (Merck Millipore, Darmstadt, Germany), a HIF1α inhibitor, for 1 hr prior to addition of 100 nM insulin for 2 more days along with the decidualization agents db‐cAMP and MPA. All the treatments were carried out under normoxic conditions. The culture media were renewed every 3 days. After treatments conditioned media were collected, and cells were harvested for RNA isolation. All the treatments have been performed at least twice.

### RNA isolation, cDNA synthesis and RT‐PCR

Total RNA was extracted, and cDNA was synthesized as described previously 32. 10 ng cDNA was subjected to RT‐PCR using TaqMan method. Ribosomal protein L13a (RPL13A) served as an internal control and was used to normalize gene expression levels. The employed TaqMan assays (Thermo Fischer Scientific) were Hs00951617_m1 for PROK1, Hs00168730_m1 for prolactin (PRL), Hs00236877_m1 for insulin‐like growth factor binding protein 1 (IGFBP1) and Hs01926559_g1 for RPL13A. The reactions were run in a StepOnePlus™ Real‐Time PCR System (Thermo Fischer Scientific), and gene expression levels were determined using the ΔΔC_T_ method. The PCR efficiency of all amplicons was 90–100%, and all determinations were performed in triplicate. PROK1 was measured in cells from six healthy volunteers.

### Enzyme‐linked immunosorbent assay

Secreted PROK1 protein produced by the decidualizing stromal cells in the presence of 0, 5, 50 or 500 nM of insulin was measured from conditioned media using enzyme‐linked immunosorbent assay (ELISA) (Abcam, Cambridge, UK) according to the manufacturer's instruction. Briefly, stromal cells were decidualized with 1 μM MPA and 0.5 mM db‐cAMP in the presence or absence of insulin for 6 days. Media and treatment were renewed after 3 days, and the conditioned media collected on the 6th day were processed for ELISA. The experiment was performed from treatments using six healthy volunteers in duplicates.

### Cell viability analysis

To assess cell viability, a MTT (3‐[4,5‐dimethylthiazol‐2‐yl]‐2,5‐diphenyl‐tetrazolium bromide) assay was used. Briefly, 10^4^ primary endometrial stromal cells were seeded in 200 μl DMEM/F12‐Glutamax supplemented with 10% HI‐FBS and 0.2% penicillin–streptomycin in a 96‐well plate. After 24 hrs, the cells were washed with PBS, and 100 μl phenol red‐free DMEM/F12 media were added with or without 50 ng/ml PROK1 (Sigma‐Aldrich), supplemented with 2% charcoal‐stripped foetal bovine serum and 0.2% penicillin–streptomycin. Cell viability was determined after 24 hrs by labelling the cells with 10 μl MTT reagent for 4 hrs. The resulting formazan crystals, which correspond to the amount of viable cells, were solubilized by the addition of 100 μl 10% sodium dodecyl sulphate solution in tris‐buffered saline (TBS). Cell viability was quantified by the measurement of the absorbance in a plate‐reader with a 595‐nm filter. Absorbance of the untreated cells was set to 100%, and the viability of treated cells was calculated accordingly. The experiments were run using cells from three healthy volunteers in quadruplicates.

### Wound healing assay

Endometrial stromal cells were seeded in 12‐well Costar plates (Sigma‐Aldrich) and cultured in DMEM/F12‐Glutamax (Thermo Fischer Scientific) supplemented with 10% HI‐FBS and 0.2% penicillin–streptomycin until confluency. The cells were scratched with a 200‐μl pipette tip, and the media were removed. The cells were washed twice with PBS to remove cell debris and 1 ml phenol red‐free DMEM/F12 media was added with or without 50 ng/ml PROK1, supplemented with 2% charcoal‐stripped foetal bovine serum and 0.2% penicillin–streptomycin. Microphotographs were taken with 40× magnification at 0 and 24 hrs with a Leica DFC420 C digital camera on (Leica Microsystems, Wetzlar, Germany) a Nikon Eclipse TS 100 inverted microscope (McCrone, Westmont, Illinois, USA). Photographs were analysed using ImageJ software (NIH, Bethesda, Maryland, USA) 37. The experiments were performed with cells from five healthy volunteers in triplicates.

To track the proliferation and migration of undifferentiated endometrial stromal cells in the presence or absence of 50 ng/ml PROK1, time‐lapse imaging was applied. Endometrial stromal cells were seeded in 24‐well Costar plates (Sigma‐Aldrich) and cultured until confluency, scratched with a 200‐μl pipette tip, washed twice with PBS and treated as described above. After the addition of the treatment, the plates were relocated to the TIRF microscope incubation chamber, at constant 37°C and under 5% CO_2_. The ZEN2 Software was used to design the experiment and guide the complete microscopic unit automatically. The time‐lapse imaging was recorded for 24 hrs, with a 15‐min. interval per capture. A field of 18 images (3 × 6), which covered a total area of 2506 × 3689.5 μm^2^, was captured using the 10× objective lens. The experiment was performed with cells from three healthy volunteers in triplicates.

### Transwell migration assay

The motility of AC‐1M88 choriocarcinoma–trophoblast hybrid cells (DSMZ, Braunschweig, Germany) was assessed using cell culture inserts with 8‐μm pores (Merck Millipore) in 24‐well plates. AC‐1M88 cells were starved for 24 hrs, and then 10^5^ cells were plated to cell culture inserts in 200 μl serum‐free F12 media with or without 50 ng/ml PROK1. 750 μl F12 media supplemented with 10% HI‐FBS was added to the lower well. After 48 hrs of incubation, inserts were washed twice with PBS, and the cells were fixed in 4% of paraformaldehyde in PBS for 15 min. The cells were stained with 0.5% crystal violet for 20 min., and the non‐migrated cells were removed with cotton swab from the upper side of the membrane. Photographs were taken with 40× magnification on a Nikon Eclipse TS 100 inverted microscope using a Leica DFC420 C digital camera. Adobe Photoshop was used to convert photographs to greyscale images, and particles were quantified by ImageJ software. The transwell migration assay was performed in quintuplicates.

### Formation of trophoblast spheroids and spheroid invasion assay

Spheroids from HTR‐8/SVneo trophoblast cells (a kind gift of Mona Nystad at the University of Tromsø, Norway) were formed on the basis of a procedure described previously 38. Briefly, for preparation of 100 spheroids, 3 × 10^5^ HTR‐8/SVneo cells were suspended in 9.11 ml of RPMI 1640 medium and mixed with 890 μl of a 2.8% methylcellulose solution (R&D Systems, Minneapolis, Minnesota, USA). In each well of a non‐adherent round‐bottom 96‐well plate, 100 μl of this suspension containing 3000 cells was seeded and incubated at 37°C in 5% CO_2_. Twenty‐four hours later, spheroids had formed and were used for the spheroid invasion assay. Spheroids were embedded using 100 μl of 6 mg/ml growth factor reduced Basement Membrane Extract (R&D Systems) diluted with serum‐free RPMI 1640 media in the presence or absence of 50 ng/ml PROK1 in round‐bottom, low‐attachment 96‐well plates and centrifuged for 5 min. at 300 *g* at 4°C. After the gel was solidified at 37°C, 100 μl complete media was added onto the top of the gel, and spheroids were incubated at 37°C in 5% CO_2_. Microphotographs were taken with 40× magnification daily for 5 days with a Leica DFC420 C digital camera on a Nikon Eclipse TS 100 inverted microscope. Photographs were analysed using ImageJ software. The spheroid invasion assay was performed in quintuplicates.

### Statistical analysis

Normality of the data was tested with the Kolgomorov–Smirnov test. Statistical analysis was performed by the one‐way anova or Friedman test followed by multiple comparisons, based on the distribution of the data, where each treatment group was tested against the untreated group. In the PI3K and HIF1α inhibitory experiments, the insulin‐treated groups were compared with their respective controls (decidual, decidual + solvent or decidual + inhibitor). Paired t‐test was used to analyse the effect of PROK1 on the viability and migration of endometrial stromal cells. In the cases of transwell migration assay and spheroid invasion assay, unpaired *t*‐test was used to analyse statistical significance between PROK1 treated and untreated samples. *P* < 0.05 was considered as significant.

## Results

### PROK1 is up‐regulated upon decidualization

To test the effect of decidualization on the expression of PROK1 in human primary endometrial stromal cells, we treated the cells with MPA, db‐cAMP or their combination for 6 days. We found that db–cAMP induced morphological transformation of the cells and up‐regulated PROK1 and the decidualization markers PRL and IGFBP1, whereas MPA did not. The combination of MPA and db‐cAMP also induced morphological transformation of the cells and enhanced up‐regulation of PROK1 and the decidualization markers PRL and IGFBP1 compared to db‐cAMP alone (Fig. S1A and B). Furthermore, we investigated the kinetics of decidualization in response to the combined use of MPA and db‐cAMP and found that decidualization was apparent after 3 days, and even more enhanced after 6 days (Fig. S1C).

### Insulin up‐regulates PROK1 in decidualizing endometrial stromal cells

**Figure 1:**
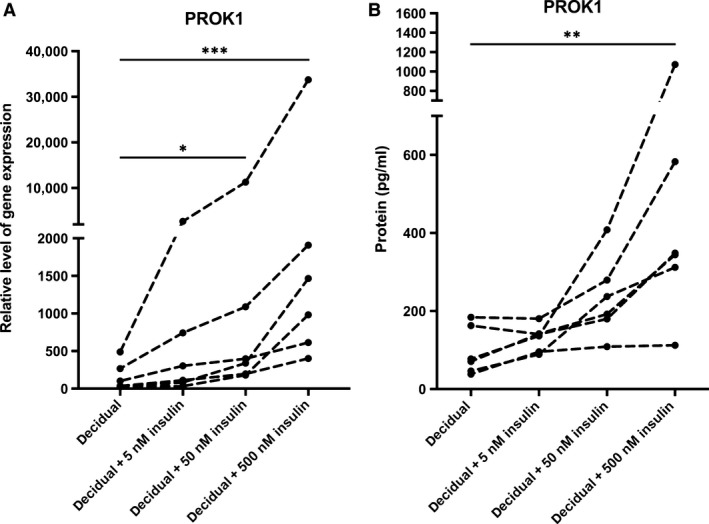
(**A**) Relative gene expression levels of PROK1 in six healthy volunteers in response to 5, 50 and 500 nM insulin in *in vitro* decidualized human endometrial stromal cells after 6 days. The values presented are medians and ranges (min–max). *P < 0.05; ***P < 0.001 in comparison to the control value. (**B**) PROK1 protein concentrations in the conditioned media in six healthy volunteers in response to 5, 50 and 500 nM insulin in *in vitro* decidualized human endometrial stromal cells after 6 days. The values presented are medians and ranges (min–max). **P < 0.01 in comparison to the control value.

Proliferative phase‐derived stromal cells were decidualized in the presence or absence of increasing doses of insulin, and relative gene expression level of PROK1 was measured. PROK1 was highly up‐regulated by insulin in a dose‐dependent fashion (ns at 5 nM, *P* < 0.05 at 50 nM, *P* < 0.001 at 500 nM) (Fig. 1A).

To verify whether insulin also regulates the protein expression of PROK1 in decidualizing endometrial stromal cells, secreted PROK1 was measured from conditioned media. The protein expression was also highly up‐regulated by insulin in a dose‐dependent manner (ns at 5 nM, ns at 50 nM, *P* < 0.01 at 500 nM) (Fig. 1B).

### PROK1 is up‐regulated by insulin *via* the induction of HIF1α

**Figure 2:**
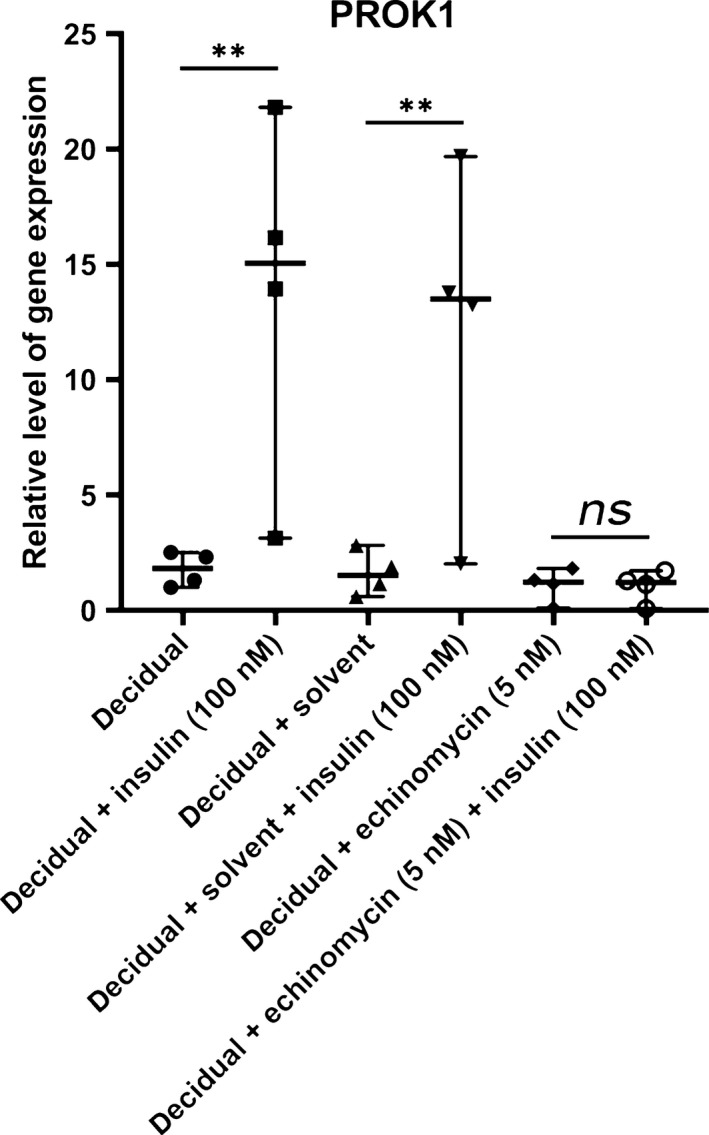
Relative gene expression levels of PROK1 in four healthy volunteers in response to 5 nM echinomycin, a HIF1α inhibitor in combination with 100 nM insulin in *in vitro* decidualized human endometrial stromal cells. The values presented are medians and ranges (min‐max). **P < 0.01 in comparison to the appropriate control value.

To test whether HIF1α is involved in the insulin‐induced up‐regulation of PROK1, we treated decidualizing stromal cells with 5 nM echinomycin, a HIF1α inhibitor, in the presence or absence of 100 nM insulin. We found that 5 nM echinomycin completely blocked insulin‐induced up‐regulation of PROK1 mRNA, meaning that insulin exerts its effect on PROK1 *via* HIF1α (Fig. 2).

### Insulin regulates PROK1 through the PI3K pathway

**Figure 3:**
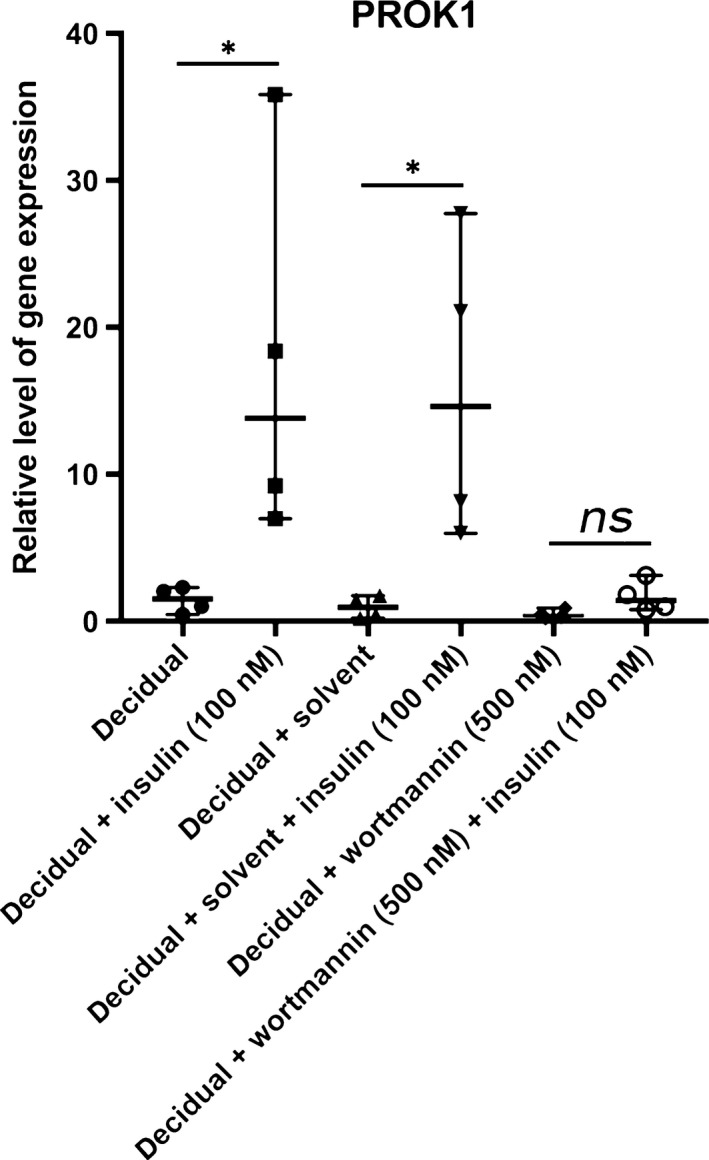
Relative gene expression levels of PROK1 in four healthy volunteers in response to 500 nM wortmannin, a PI3K inhibitor in combination with 100 nM insulin in *in vitro* decidualized human endometrial stromal cells. The values presented are medians and ranges (min‐max). *P < 0.05 in comparison to the appropriate control value.

To investigate whether insulin regulates PROK1 through the PI3K pathway, we blocked PI3K signalling with 500 nM wortmannin during the decidualization process in the presence or absence of insulin. PI3K inhibition resulted in an almost complete inhibition of insulin‐induced up‐regulation of PROK1 (Fig. 3). This suggests that the regulation of insulin on PROK1 is conveyed by the PI3K signalling pathway.

### PROK1 does not influence viability of endometrial stromal cells

**Figure 4:**
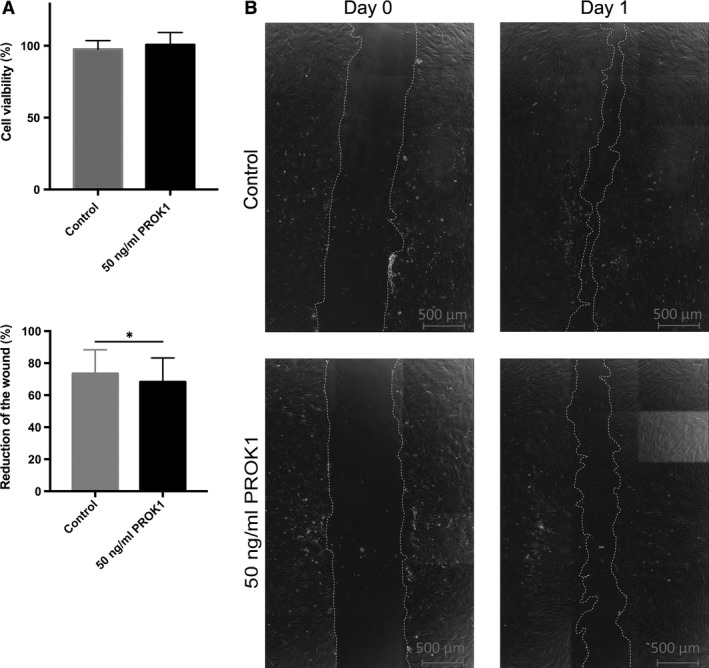
Cell viability and wound healing assay using human primary endometrial stromal cells with or without 50 ng/ml PROK1. (**A**) Cell viability in the presence or absence of 50 ng/ml PROK1 for 24 hrs using cells from three healthy volunteers. Values are presented as means and standard deviations. (**B**) Representative microphotographs (40× magnification) of wound healing assay were taken with live‐cell TIRF microscopy imaging. (**C**) Reduction of the wound was analysed by ImageJ software. Values are presented as means and standard deviations. *P < 0.05.

To test whether PROK1 has an effect on cell viability, i.e. proliferation and cell death, MTT assay was performed on undifferentiated endometrial stromal cells. PROK1 had no significant effect on the cell viability in a 24‐hrs experiment compared to untreated controls (Fig. 4A).

### PROK1 inhibits migration of endometrial stromal cells

To investigate whether PROK1 has an effect on the migration of endometrial stromal cells, we performed a wound healing assay on the undifferentiated endometrial stromal cells. Treatment with 50 ng/ml PROK1 slightly, but significantly reduced the migration of endometrial stromal cells after 24 hrs (*P* < 0.05) (Fig. 4B and C and Video S1 and S2).

### PROK1 inhibits migration of AC‐1M88 trophoblast–choriocarcinoma cells

**Figure 5:**
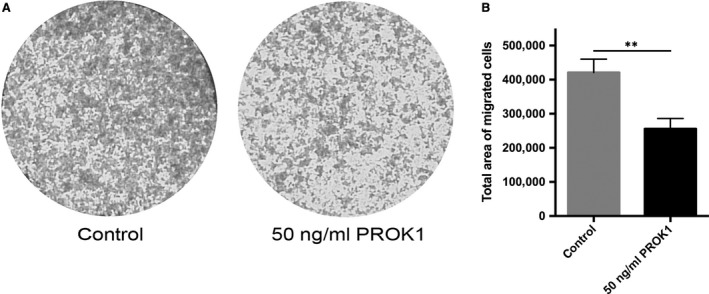
Transwell migration assay using AC‐1M88 choriocarcinoma–trophoblast cells with or without 50 ng/ml PROK1 (**A**) Representative microphotographs (40× magnification) were taken with a Leica DFC420 C digital camera on a Nikon Eclipse TS 100 inverted microscope and converted to greyscale image by Adobe Photoshop. (**B**) Total area of the migrated cells was analysed by ImageJ software. Values are presented as means and standard deviations. **P < 0.01.

To demonstrate the effect of PROK1 on the migration of AC‐1M88 trophoblast–choriocarcinoma cells, we performed a transwell migration assay in the absence or presence of 50 ng/ml PROK1. We found that PROK1 significantly inhibited the migration of AC‐1M88 cells (*P* < 0.01) (Fig. 5A and B).

### PROK1 inhibits invasion of HTR‐8/SVneo trophoblast spheroids

**Figure 6:**
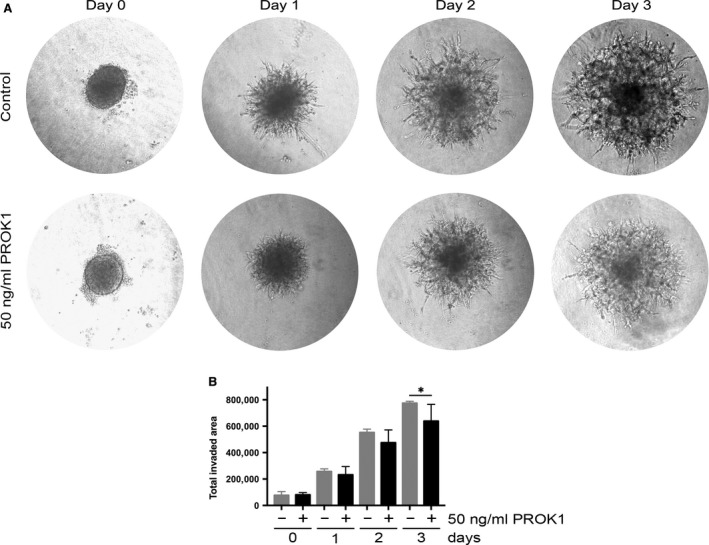
Spheroid invasion assay using HTR‐8/SVneo trophoblast cells with or without 50 ng/ml PROK1. (**A**) Representative microphotographs (40× magnification) were taken with a Leica DFC420 C digital camera on a Nikon Eclipse TS 100 inverted microscope and converted to greyscale image by Adobe Photoshop. (**B**) Total invaded area analysed by ImageJ software. Values are presented as means and standard deviations. *P < 0.05.

Spheroids were formed using HTR‐8/SVneo cells and embedded into basement membrane extract in the presence or absence of 50 ng/ml PROK1. The area covered by individual spheroids was determined daily. We found that invasion of the spheroids was reduced by PROK1, which reached significance in comparison to controls after 3 days (*P* < 0.05) (Fig. 6A and B).

## Discussion

This study is the first to show that insulin up‐regulates gene and protein expression of the pro‐angiogenic factor PROK1 in decidualizing human endometrial stromal cells. We furthermore demonstrated that insulin enhances PROK1 expression *via* HIF1α, a well‐known regulator of PROK1, and that the PI3K pathway is involved in insulin‐induced up‐regulation of PROK1. It was also shown here for the first time that PROK1 does not affect viability, however decreases migration of endometrial stromal cells, a process that is crucial for adequate embryo implantation. In addition, it was demonstrated that PROK1 restricts the migration of AC‐1M88 trophoblast–choriocarcinoma hybrid cells, as well as the invasion of HTR‐8/SVneo trophoblast spheroids.

In this study, we demonstrated that PROK1 gene and protein expression are up‐regulated during *in vitro* decidualization, and this is in agreement with the *in vivo* findings in human endometrium by others 2. This up‐regulation in decidualizing human endometrial stromal cells was even more enhanced by insulin in a dose‐dependent manner. It must be noted that PROK1 gene expression level after *in vitro* decidualization showed high variety among the volunteers, and two volunteers showed remarkably higher inducibility of PROK1 in response to insulin. These variances might be due to genetic polymorphisms of PROK1 gene, as certain polymorphisms have been associated with different gene expression levels of PROK1 39.

In the present study, we demonstrated that when decidualizing endometrial stromal cells were treated with a HIF1α inhibitor or a PI3K inhibitor under normoxic conditions, they both prevented insulin‐induced up‐regulation of PROK1. These findings are in agreement with previous publications on the ability of HIF1α to up‐regulate PROK1 in other cell types 5, 10. Our data also support previous findings on the possible effect of insulin on HIF1α and the involvement of the PI3K pathway in this process and suggest that insulin up‐regulates PROK1 in decidualizing endometrial stromal cells *via* PI3K‐mediated HIF1α induction 13, 14, 15, 16, 17.

We also demonstrated that PROK1 reduced the migratory capacity of endometrial stromal cells. It was previously reported that invasion of the embryonic trophoblast required motility of maternal endometrial stromal cells 38, 40, 41, 42. Moreover, endogenous PROK1 may act in an autocrine manner in decidualizing endometrial stromal cells *via* the prokineticin receptor 1 (PROKR1) 43. We speculate that PROK1 dysregulation may interfere with normal implantation by inhibiting the migration of endometrial stromal cells. We also demonstrated that PROK1 did not affect the viability, i.e. proliferation, cell death and metabolism of endometrial stromal cells significantly. Furthermore, the negligible enhancement by PROK1 on cell viability can be resulted in an underestimation of restriction of migration of endometrial stromal cells by PROK1.

In this study, we could show that PROK1 significantly inhibits the migration of AC‐1M88 trophoblast–choriocarcinoma cells. As wound healing assay proved to be challenging with AC‐1M88 cells, because of the detachment of the whole cell layer when scratching, we performed transwell migration assay. We also demonstrated that invasion of HTR‐8/SVneo spheroids is restricted by PROK1. It was previously shown that PROK1 inhibits migration of HTR‐8/SVneo cells, invasion of placental villous explants and tube‐like organization of HTR‐8/SVneo cells 6. The authors suggested that sustained PROK1 expression beyond 10–12 weeks of gestation might cause an inhibition of deeper invasion of the decidua by differentiating extravillous trophoblasts that might consequently lead to a failure in maternal spiral artery remodelling 6. Furthermore, it was recently demonstrated that sustained PROK1 levels beyond the first trimester of gestation cause defects in placental organization and function, increase hypoxia and decrease trophoblast invasion in mice, leading to gestational hypertension and dysregulated maternal kidney function, typical symptoms of pre‐eclampsia 44. Our results further support the previous suggestion that PROK1 may restrict the migration and invasion of embryonic trophoblast cells into maternal decidua 6.

Metabolic disorders such as PCOS, obesity and insulin resistance are associated with elevated risk of both miscarriage, including recurrent pregnancy loss 19, 21, 22, 23, 27, 28 and pre‐eclampsia 18, 30. Furthermore, a direct relationship was suggested between hyperinsulinaemia—a condition that can be present in these metabolic disorders—and early pregnancy loss/recurrent miscarriage 20, 23, 26, 27, 31, as well as pre‐eclampsia 24, 25, 29.

Based on our data, we hypothesize that maternal hyperinsulinaemia could increase decidual PROK1 expression that might lead to impaired invasion of extravillous trophoblasts in the first trimester of the pregnancy. Clinical studies have shown that certain polymorphisms of PROK1 and PROKR1 are associated with altered susceptibility for recurrent pregnancy loss 39, 45, 46. It was also demonstrated that gene expression level of PROK1 is higher in decidualizing stromal cells from patients with recurrent pregnancy loss compared to controls. This could result in prolonged endometrial receptivity, i.e. a temporally unique sequence of factors that make the endometrium receptive to embryo attachment and implantation, allowing out‐of‐phase implantation in an unsupportive uterine environment 47, 48, 49.

A line of evidence suggests that impaired invasion of extravillous trophoblasts might trigger a number of events. The shallow invasion of extravillous trophoblasts in the first trimester of pregnancy was shown to impair remodelling of the maternal spiral arteries that was associated with foetal growth restriction and pre‐eclampsia 50, 51, resulting in a relatively hypoxic milieu in the placenta 7, 52. The relative placental hypoxia was demonstrated to dysregulate several angiogenic factors including VEGF 53. It was shown that these factors are dysregulated, and branching of the placental vascularization is enhanced in pre‐eclampsia 54, 55, suggesting that switch from branching to non‐branching angiogenesis is disturbed in this pregnancy complication. PROK1 was shown to enhance tube‐like formation of microvascular placental endothelial cells (HPEC), suggesting that PROK1 contributes to branching of the vascular tree 56. Furthermore, maternal serum level of PROK1 was shown to be significantly higher in pre‐eclampsia and foetal growth restriction than in normal pregnancies 4, 6.

Taken together, our data show for the first time that PROK1—a crucial factor in pregnancy complications such as recurrent pregnancy loss, pre‐eclampsia, foetal growth restriction and spontaneous preterm birth—is up‐regulated by insulin in decidualizing endometrial stromal cells. We demonstrate that PROK1 negatively influences three processes, which are suggested to be essential for successful implantation, placenta development and normal maintenance of the pregnancy, i.e. migration of endometrial stromal cells and migration and invasion of trophoblasts. Our results support the previous suggestions that hyperinsulinaemia might play a role in the mechanisms behind hypoxia‐related pregnancy complications such as miscarriage and pre‐eclampsia.

## Conflict of interest

The authors declare no conflict of interests.

## Supporting information


**Figure S1** A. Representative micrographs of undifferentiated stromal cells and cells in response to MPA (1 μM), db‐cAMP (0.5 mM) and their combined treatment after 6 days were taken using an inverted microscope with 40× magnification. B. Relative gene expression levels of PRL, IGFBP1 and PROK1 based on three healthy volunteers in response to MPA (1 μM), db‐cAMP (0.5 mM) and their combined treatment in endometrial stromal/decidualizing cells after 6 days. The values presented are medians and ranges (min‐max). *P < 0.05 and **P < 0.01 in comparison to the control (stromal) value. C. Relative gene expression levels of PRL, IGFBP1 and PROK1 based on three healthy volunteers in response to decidualization agents MPA (1 μM) and db‐cAMP (0.5 mM) in endometrial stromal/decidual cells after 0, 3 and 6 days. The values presented are medians and ranges (min‐max). *P < 0.05, **P < 0.01 and ***P < 0.001 in comparison to the control (stromal) value.

**Video S1** Representative time‐lapse imaging on the migration of endometrial stromal cells recorded for 24 hrs. The experiment was performed in three healthy volunteers.

**Video S2** Representative time‐lapse imaging on the migration of endometrial stromal cells in the presence of 50 ng/ml PROK1 recorded for 24 hrs. The experiment was performed in three healthy subjects.
