# Supplementary figures and images for: Prokineticin 1 is up‐regulated by insulin in decidualizing human endometrial stromal cells
Source: J Cell Mol Med. 2017 Aug 7;22(1):163–72. doi: 10.1111/jcmm.13305 (PMC5742737; doi:10.1111/jcmm.13305)

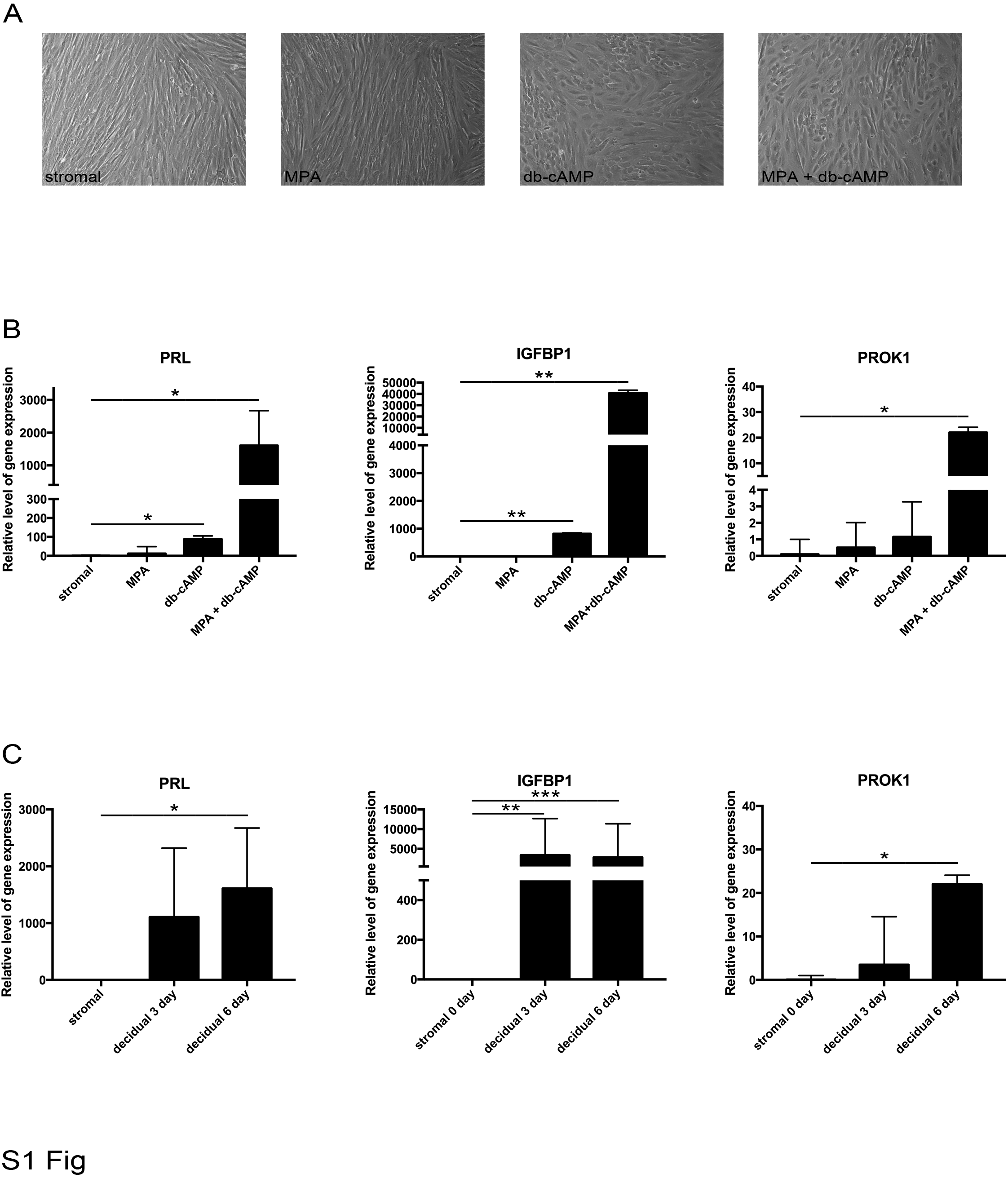

Supplement: Supplementary file 1 — Figure S1 A. Representative micrographs of undifferentiated stromal cells and cells in response to MPA (1 μM), db‐cAMP (0.5 mM) and their combined treatment after 6 days were taken using an inverted microscope with 40× magnification. B. Relative gene expression levels of PRL, IGFBP1 and PROK1 based on three healthy volunteers in response to MPA (1 μM), db‐cAMP (0.5 mM) and their combined treatment in endometrial stromal/decidualizing cells after 6 days. The values presented are medians and ranges (min‐max). *P < 0.05 and **P < 0.01 in comparison to the control (stromal) value. C. Relative gene expression levels of PRL, IGFBP1 and PROK1 based on three healthy volunteers in response to decidualization agents MPA (1 μM) and db‐cAMP (0.5 mM) in endometrial stromal/decidual cells after 0, 3 and 6 days. The values presented are medians and ranges (min‐max). *P < 0.05, **P < 0.01 and ***P < 0.001 in comparison to the control (stromal) value. [file JCMM-22-163-s001.tif]
